# Elevated first-trimester hepcidin level is associated with reduced risk of iron deficiency anemia in late pregnancy: a prospective cohort study

**DOI:** 10.3389/fnut.2023.1147114

**Published:** 2023-08-15

**Authors:** Peng Sun, Yueqin Zhou, Suhua Xu, Xiaotong Wang, Xiuxiu Li, Hailin Li, Zongyu Lin, Fenglian Huang, Lewei Zhu, Yanna Zhu

**Affiliations:** ^1^Shenzhen Nanshan Maternity and Child Healthcare Hospital, Shenzhen, China; ^2^Department of Maternal and Child Health, School of Public Health, Sun Yat-sen University, Guangzhou, China

**Keywords:** hepcidin, iron deficiency, anemia, pregnancy, hemoglobin

## Abstract

**Background:**

Iron deficiency (ID) and iron deficiency anemia (IDA) during pregnancy are highly prevalent worldwide. Hepcidin is considered an important biomarker of iron status. Currently, few longitudinal cohort studies have assessed the potential causal relationship between hepcidin and ID/IDA. Therefore, we aimed to investigate the association of first-trimester maternal serum hepcidin with third-trimester ID/IDA risk in a prospective cohort.

**Methods:**

Total of 353 non-ID/IDA pregnant women at 11–13 weeks’ gestation were enrolled in Southern China and followed up to 38 weeks of gestation. Data on demography and anthropometry were obtained from a structured questionnaire at enrollment. Iron biomarkers including hepcidin were measured at enrollment and follow-up. Regression models were used to evaluate the association of first-trimester hepcidin with third-trimester ID/IDA risk.

**Results:**

Serum hepcidin levels substantially decreased from 19.39 ng/mL in the first trimester to 1.32 ng/mL in the third trimester. Incidences of third-trimester ID and IDA were 46.2 and 11.4%, respectively. Moreover, moderate and high levels of first-trimester hepcidin were positively related to third-trimester hepcidin (log-transformed *β* = 0.51; 95% CI = 0.01, 1.00 and log-transformed *β* = 0.66; 95% CI = 0.15, 1.17). Importantly, elevated first-trimester hepcidin was significantly associated with reduced risk of third-trimester IDA (OR = 0.38; 95% CI = 0.15, 0.99), but not with ID after adjustment with potential confounders.

**Conclusion:**

First-trimester hepcidin was negatively associated with IDA risk in late pregnancy, indicating higher first-trimester hepcidin level may predict reduced risk for developing IDA. Nonetheless, given the limited sample size, larger studies are still needed.

## 1. Introduction

Iron deficiency (ID), and specifically iron deficiency anemia (IDA), has been well-recognized as one of the most common nutritional deficiencies during pregnancy globally (1). The prevalence of gestational anemia was reported to be 26% in developed regions such as the United States and Europe and reached 46%–48% in Southeast Asia and Africa (2). According to the WHO, 50% of anemia is attributed to ID (3). In China, the prevalence of maternal ID and IDA was reported to be 42.6% and 19.1%, respectively (4). Numerous studies have shown that antenatal ID and IDA are associated with increased risk of maternal mortality, preterm birth, small for gestational age, low birth weight, and long-term influence on cognitive function in the offspring (3, 5–7). Thus, it is essential to identify the risks of ID and IDA early.

Although there are some well-established methods to detect iron status, accurate identification of maternal ID and IDA with conventional markers still remains a challenge (8). Serum ferritin (SF) and hemoglobin (Hb) serve as common markers of ID and IDA. Hb is a sensitive indicator of ID, because IDA, as a type of Hb disorder, directly results from ID. However, Hb in the diagnosis of ID/IDA may be interfered with other Hb disorders such as thalassemia. Moreover, SF can be confounded by inflammation and infection (9). Therefore, a biomarker that enables accurate identification of the risk of iron status is needed.

Hepcidin, a cysteine-rich antimicrobial peptide hormone secreted by the liver, has been shown in previous studies that it exerts a crucial role in iron metabolism (10). Although both hepcidin and SF are correlated with inflammation, such as C-reactive protein (CRP), prior studies have shown that hepcidin is closely related to iron status during pregnancy (11–13). In detail, evidence from the studies have shown that the level of hepcidin in pregnant women with IDA was lower than that in controls (14, 15). Some cohort studies also revealed the significant association between hepcidin and iron status, but mostly within a single time period (16, 17). Furthermore, only one sub-analysis of a pooled dataset among pregnant women before 28 weeks’ gestation found that baseline serum hepcidin >1.6 μg/L was associated with a reduced risk of IDA at delivery (11). Additionally, hepcidin is suppressed in the second and third trimester, especially the level of hepcidin was extremely low and hard to check out in the third trimester (18). Therefore, it is necessary to explore the hepcidin level at early pregnant stage with iron status in late pregnancy.

Our objectives were to investigate the associations of serum hepcidin levels in the first trimester with iron status and ID/IDA risk in the third trimester through a prospective cohort study, thus providing scientific evidence for identification of ID/IDA risk at an early stage.

## 2. Materials and methods

### 2.1. Study design and participants

This prospective cohort study was conducted among pregnant women at Shenzhen Nanshan Maternity and Child Healthcare Hospital in Shenzhen city, South China, from May 2019 to April 2020. Briefly, non-ID/IDA women with singleton pregnancy, aged 18 to 45 years, were recruited between 11 and 13 weeks of gestation (here non-ID/IDA was defined as the absence of SF < 20 ng/mL or Hb < 110 g/L). Any subjects with (1) a history of iron-supplementation intake during the past 3 months; (2) a history of hypertension and diabetes; or (3) unable to complete questionnaire or refused to sign the informed consent, were excluded. Written informed consents were obtained from all participants at the time of recruitment. The participants were invited to complete a structured questionnaire, undergo physical examinations, and provide blood samples after enrollment and were recalled for blood samples collection at 38 weeks of gestation. This study was approved by the Ethics Committee of School of Public Health Sun Yat-sen University, and conducted corresponding to the Declaration of Helsinki.

### 2.2. Laboratory tests

Each pregnant woman who met the eligible criteria had her venous blood drawn by a trained nurse in the morning after overnight fasting in the first trimester (12.14 ± 0.04 gestational weeks) and the third trimester (38.62 ± 0.13 gestational weeks), in accordance with the standard protocol. Serum iron biomarkers including serum hepcidin, SF, Hb and serum iron (SI), and inflammatory biomarkers like CRP were tested within 2 h by trained technicians in the hospital laboratory. Serum hepcidin levels were measured using a commercially available quantikine ELISA kit (DHP250; R&D system; USA) according to the manufacturer’s instructions, which is a validated and highly sensitive enzyme immunoassay for quantitative *in vitro* diagnostic determination of hepcidin in human cell culture supernate, serum, plasma or urine. SF concentrations were determined by the enzyme immunoassay method, using commercial kits (FERRITIN ELISA; Diametra, Boldon, UK). Hb levels were quantified on an automated hematological analyser (TC Hemaxa 1,000; Teco Diagnostic, Anaheim, CA, USA), using a hemiglobin-cyanide method. SI levels were assessed by a commercial test, using a colorimetric method (Ferentest, bioMérieux® SA, France). Serum concentrations of CRP were determined using an immunoturbidimetric assay. For these parameters, the intra-assay and inter-assay CV (%) were below 5.7 and 6.7%, respectively. The remaining blood samples were stored at˗80 C until assayed.

### 2.3. Exposure assessment

The exposure was serum hepcidin level in the first trimester, which was divided into tertiles based on its distribution among all participants. First-trimester serum hepcidin was classified into three categories: (1) low serum hepcidin, ≤11.85 ng/mL (reference); (2) moderate serum hepcidin, 11.86–27.43 ng/mL; and (3) high serum hepcidin, ≥27.44 ng/mL.

### 2.4. Assessment of outcomes

The primary outcomes were the incidences of ID and IDA in the third trimester. ID and IDA were classified according to SF and Hb. ID was defined as SF < 20 ng/mL and IDA was defined as ID plus low Hb (Hb < 110 g/L), in accordance with the definition proposed by Chinese Medical Association, 2014 (19). The secondary outcomes were iron status in the third trimester, including serum hepcidin, SF, Hb and SI.

### 2.5. Assessment of covariates

Data on demographic information and pregnancy history were collected from a structured questionnaire at enrollment, including maternal age (years), educational level, monthly income, participant source, gestational age, gravidity and parity. Anthropometric data at enrollment were obtained by experienced clinicians and nurses. Barefoot height was measured to the nearest 0.1 cm using a stadiometer (Yilian TZG, Jiangsu, PRC), and body weight was measured to the nearest 0.1 kg with a self-zeroing scale (Hengxing TGT-140, Jiangsu, PRC). Pre-pregnancy weight was measured and recorded in health booklets by professional staffs during the pre-pregnancy checkups. Pre-pregnancy BMI (pre-BMI) was calculated by dividing one’s pre-pregnancy weight in kilograms by her height in meters and categorized as underweight (<18.5 kg/m2), normal (18.5–23.9 kg/m2), or overweight (≥24 kg/m2), according to Chinese criteria (20). Intake of iron supplementation during pregnancy was obtained from medical records.

### 2.6. Statistical analysis

Normality was assessed using the Shapiro–Wilk test and Q-Q plot. Data were presented as mean ± standard deviation or median (inter-quartile range) for continuous variables and number (percentage) for categorical variables. The difference among three groups were compared by One-way ANOVA (continuous variables with normal distribution), or Kruskal-Wallis H tests (continuous variables with skewed distribution), and Chi-square tests (categorical variables). We used linear regression model to evaluate the associations of first-trimester serum hepcidin with third-trimester iron biomarkers (serum hepcidin, SF, Hb and SI) and inflammatory biomarker (CRP). Right-skewed biomarkers including serum hepcidin, SF, SI and CRP in the third trimester, as dependent variables, were log(e)-transformed to normalize distributions prior to linear regression analysis. Resulting regression coefficients (*β*) expressed the change in log-transformed biomarker levels that are associated with moderate or high tertiles of first-trimester hepcidin, compared to low tertile of first-trimester hepcidin. In addition, logistic regression models were built to examine the associations between first-trimester serum hepcidin and third-trimester ID/IDA risk. Maternal age, pre-pregnancy BMI, parity, iron supplementation during pregnancy, and CRP or serum hepcidin in the third trimester were included as covariates in the regression models. All statistical analyses were performed using R 4.0; *p* < 0.05 was considered significant.

## 3. Results

### 3.1. Characteristics of the participants stratified by tertiles of first-trimester serum hepcidin

The flow chart of the study participants is shown in Supplementary Figure S1. Of the total 353 pregnant women recruited, 264 participants with full data were included in the final analysis. The characteristics of the participants stratified by tertiles of first-trimester serum hepcidin are presented in Table 1. The mean age of participants was 29.12 ± 4.45 years and the mean gestational age was 12.15 ± 0.64 weeks at enrollment. The median serum hepcidin concentration was 19.39 (9.69–33.59) ng/ml in the first trimester and 1.32 (0.52–6.67) ng/ml in the third trimester. In the first trimester, no significant differences were observed in demographic characteristics among pregnant women (*p* > 0.05), and significant differences were observed only in SF levels (*p* < 0.05). In the third trimester, significantly higher serum hepcidin, Hb and SI levels were observed among pregnant women with higher tertiles of first-trimester hepcidin compared to those with the lowest tertile (*p* < 0.05). In addition, 122 (46.2%) incident ID cases and 30 (11.4%) incident IDA cases were identified in the third trimester, respectively.

**Table 1:** Characteristics of individuals stratified by tertiles of first-trimester serum hepcidin levels.

Characteristics	Total (*N* = 264)	Low hepcidin	Moderate hepcidin	High hepcidin	*P*
		≤ 11.85 (*N* = 89)	11.86–27.43 (*N* = 87)	≥ 27.44 (*N* = 88)	
Age, years	29.12 ± 4.45	29.02 ± 4.61	29.07 ± 4.35	29.27 ± 4.43	0.924
Educational level					
College or lower	74 (41.3)	23 (39.7)	26 (44.1)	25 (40.3)	0.871
University or higher	105 (58.7)	35 (60.3)	33 (55.9)	37 (59.7)	
Monthly income					
≤5,000 RMB	89 (50.0)	27 (46.6)	32 (55.2)	30 (48.4)	0.618
>5,000 RMB	89 (50.0)	31 (53.4)	26 (44.8)	32 (51.6)	
Participant source					
Rural	202 (76.5)	65 (73.0)	67 (77.0)	70 (79.5)	0.588
Urban	62 (23.5)	24 (27.0)	20 (23.0)	18 (20.5)	
Gestational age at enrolment, weeks	12.15 ± 0.64	12.14 ± 0.61	12.20 ± 0.66	12.11 ± 0.65	0.684
Gravidity					
1	86 (32.6)	23 (25.8)	26 (29.9)	37 (42.0)	0.057
≥2	178 (67.4)	66 (74.2)	61 (70.1)	51 (58.0)	
Parity					
Primiparous	112 (42.4)	33 (37.1)	33 (37.9)	46 (52.3)	0.072
Multiparous	152 (57.6)	56 (62.9)	54 (62.1)	42 (47.7)	
Height, cm	158.48 ± 5.18	158.84 ± 4.28	158.15 ± 6.48	158.42 ± 4.59	0.674
Weight at enrolment, kg	53.23 ± 8.23	53.62 ± 9.02	53.73 ± 7.34	52.48 ± 8.42	0.710
Pre-BMI, kg/m^2^	21.31 ± 2.76	21.28 ± 2.81	21.47 ± 2.92	21.18 ± 2.54	0.781
Pre-BMI category					
Normal	189 (71.6)	63 (70.8)	63 (72.4)	63 (71.6)	0.870
Underweight	35 (13.3)	13 (14.6)	9 (10.3)	13 (14.8)	
Overweight	40 (15.2)	13 (14.6)	15 (17.2)	12 (13.6)	
Iron supplementation during pregnancy					
Received	59 (22.3)	25 (28.1)	19 (21.8)	15 (17.0)	0.209
Not received	205 (77.7)	64 (71.9)	68 (78.2)	73 (83.0)	
First trimester					
SH, ng/mL	19.39 (9.69–33.59)	8.03 (5.01–9.76)	19.43 (16.14–23.84)^a^	40.74 (33.76–58.53)^a^	<0.001*
SF, ng/mL	85.00 (54.85–124.00)	52.00 (41.00–80.75)	86.00 (61.50–116.00)^a^	123.00 (93.75–175.25)^a^	<0.001*
Hb, g/L	126.21 ± 8.08	125.31 ± 7.26	126.61 ± 8.31	126.72 ± 8.63	0.473
SI, umol/L	21.07 (17.32–25.06)	20.30 (16.76–24.09)	22.43 (17.88–25.73)	20.41 (17.28–25.55)	0.128
Third trimester					
SH, ng/mL	1.32 (0.52–6.67)	0.85 (0.37–3.25)	1.47 (0.67–7.94)	2.31 (0.76–7.48)^a^	0.007*
SF, ng/mL	20.00 (14.15–31.00)	20.00 (13.00–27.10)	20.00 (13.25–29.50)	20.85 (16.00–36.50)	0.201
Hb, g/L	118.17 ± 14.40	115.28 ± 14.44	120.79 ± 13.04^a^	118.50 ± 15.25	0.038*
SI, umol/L	12.90 (9.80–17.56)	12.73 (8.60–16.48)	13.82 (11.32–18.60)^a^	12.90 (9.38–16.97)	0.041*
CRP, mg/L	6.85 (3.77–15.47)	6.85 (3.67–11.83)	6.85 (3.50–14.30)	6.88 (4.73–26.91)	0.211
ID	122 (46.2)	43 (48.3)	41 (47.1)	38 (43.2)	0.774
IDA	30 (11.4)	16 (18.0)	7 (8.0)	7 (8.0)	0.054

RMB, Renminbi; pre-BMI: pre-pregnancy body mass index; SH, serum hepcidin; SF, serum ferritin; Hb, hemoglobin; SI, serum iron; CRP, C-reactive protein; ID, iron deficiency; IDA, iron deficiency anemia.

Data were presented as N (percentage) for categorical variables and mean ± standard deviation or median (inter-quartile range) for normal or skewed continuous variables.

* *p* < 0.05 among three groups, assessed by Chi-square test for categorical variables and one-way ANOVA and Kruskal-Wallis H test for continuous variables when appropriate. Post-hoc tests with Bonferroni.

a *p* < 0.05, compared to low hepcidin group.

### 3.2. The relationships between tertiles of first-trimester serum hepcidin and third-trimester iron status

We evaluated the relationships between tertiles of first-trimester serum hepcidin and third-trimester iron status (Table 2). Significantly higher serum hepcidin levels in the third trimester were more likely to be observed in pregnant women with moderate first-trimester hepcidin (log-transformed *β* = 0.51; 95% confidence interval [CI] = 0.01, 1.00), as well as those with high first-trimester hepcidin (log-transformed *β* = 0.66; 95% CI = 0.15, 1.17), compared to those with low first-trimester hepcidin, after adjusting for maternal age, pre-pregnancy BMI, parity, iron supplementation during pregnancy and CRP. These β values, when calculated back to original scales of third-trimester serum hepcidin, mean that women with moderate and high first-trimester hepcidin, respectively, had 66.5% (i.e., [e^0.51^–1] × 100%) and 93.5% (i.e., [e^0.66^–1] × 100%) higher levels of serum hepcidin in the third trimester, compared to those with low first-trimester hepcidin. Moreover, in comparison to low first-trimester hepcidin, moderate first-trimester hepcidin was positively associated with SI (log-transformed *β* = 0.20; 95% CI = 0.06, 0.34) and Hb (*β* = 5.43; 95% CI = 1.17, 9.70) in the third trimester, after adjustment with the mentioned covariates. Similarly, these β values mean that women with moderate first-trimester hepcidin had 22.1% (i.e., [e^0.20^–1] × 100%) and 5.43 ng/mL higher levels of SI and Hb in the third trimester than those with low first-trimester hepcidin, respectively. However, no significant associations were observed between first-trimester hepcidin and SF or CRP in the third trimester.

**Table 2:** The associations between first-trimester serum hepcidin levels and third-trimester iron status.

Outcomes	Low hepcidin	Moderate hepcidin		High hepcidin	
	≤11.85 (*N* = 89)	11.86–27.43 (*N* = 87)	*P*	≥27.44 (*N* = 88)	*P*
SH^†^					
Crude	Reference	0.54 (0.03, 1.04)	0.038*	0.73 (0.22, 1.23)	0.005*
Adjusted^‡^	Reference	0.51 (0.01, 1.00)	0.046*	0.66 (0.15, 1.17)	0.011*
SF^†^					
Crude	Reference	0.02 (−0.23, 0.27)	0.857	0.19 (−0.06, 0.44)	0.145
Adjusted^‡^	Reference	0.01 (−0.24, 0.26)	0.958	0.17 (−0.08, 0.42)	0.183
Hb					
Crude	Reference	5.51 (1.27, 9.75)	0.011*	3.22 (−1.01, 7.45)	0.135
Adjusted^‡^	Reference	5.43 (1.17, 9.70)	0.013*	2.98 (−1.33, 7.30)	0.175
SI^†^					
Crude	Reference	0.20 (0.06, 0.34)	0.006*	0.04 (−0.10, 0.19)	0.532
Adjusted^‡^	Reference	0.20 (0.06, 0.34)	0.005*	0.03 (−0.11, 0.17)	0.637
CRP^†^					
Crude	Reference	0.15 (−0.28, 0.57)	0.489	0.34 (−0.08, 0.77)	0.110
Adjusted^§^	Reference	0.12 (−0.30, 0.54)	0.579	0.41 (−0.02, 0.84)	0.063

SH, serum hepcidin; SF, serum ferritin; Hb, hemoglobin; SI, serum iron; CRP, C-reactive protein.

Data were presented as β (95% confidence interval).

* *p* < 0.05, estimated by liner regression model.

† The dependent varibles were log(e)-transformed prior to linear regression analysis. Thus, the β values expressed the change in log-transformed biomarker levels that are associated with moderate or high tertiles of first-trimester hepcidin, compared to low tertile of first-trimester hepcidin.

‡ Adjusted for maternal age, pre-pregnancy BMI, parity, iron supplementation during pregnancy and CRP in the third trimester.

§ Adjusted for maternal age, pre-pregnancy BMI, parity, iron supplementation during pregnancy and hepcidin in the third trimester.

### 3.3. The relationships between tertiles of first-trimester serum hepcidin and ID/IDA risk in the third trimester

Table 3 shows the relationships between tertiles of first-trimester serum hepcidin and ID/IDA risk in the third trimester. In the unadjusted analyses, when compared to low first-trimester hepcidin, moderate and high first-trimester hepcidin was marginally associated with reduced risk of IDA in the third trimester (crude odds ratio [OR] = 0.40; 95% CI: 0.16, 1.03 and crude OR = 0.39; 95% CI: 0.15, 1.01, respectively). Furthermore, after additional adjustment with maternal age, pre-pregnancy BMI, parity, iron supplementation during pregnancy and CRP, first-trimester hepcidin was independently inversely associated with IDA risk in the third trimester (moderate vs. low hepcidin: adjusted OR = 0.38; 95% CI: 0.15, 0.99 and high vs. low hepcidin: adjusted OR = 0.38; 95% CI: 0.14, 0.99). However, when extreme tertiles were compared, first-trimester hepcidin was not substantially related to ID risk in the third trimester regardless of whether the selected confounders were controlled for.

**Table 3:** The associations between first-trimester serum hepcidin levels and third-trimester iron deficiency and iron deficiency anemia.

Outcomes	Low hepcidin	Moderate hepcidin		High hepcidin	
	≤11.85 (*N* = 89)	11.86–27.43 (*N* = 87)	*P*	≥27.44 (*N* = 88)	*P*
ID, *n* (%)	43 (48.3)	41 (47.1)			38 (43.2)		
Crude	Reference	0.95 (0.53, 1.72)	0.875	0.81 (0.45, 1.47)	0.493
Adjusted^†^	Reference	0.98 (0.54, 1.80)	0.950	0.82 (0.44, 1.51)	0.521
IDA, *n* (%)	16 (18.0)	7 (8.0)		7 (8.0)	
Crude	Reference	0.40 (0.16, 1.03)	0.056	0.39 (0.15, 1.01)	0.053
Adjusted^†^	Reference	0.38 (0.15, 0.99)	0.047*	0.38 (0.14, 0.99)	0.049*

ID, iron deficiency; IDA, iron deficiency anemia.

Data were presented as odds ratio (95% confidence interval).

* *P* < 0.05, estimated by logistic regression model.

† Adjusted for maternal age, pre-pregnancy BMI, parity, iron supplementation during pregnancy and CRP in the third trimester.

## 4. Discussion

The prospective associations between maternal serum hepcidin in early pregnancy and subsequent risks of ID and IDA are still unknown. Our longitudinal cohort study showed that elevated first-trimester serum hepcidin was associated with increased serum hepcidin and higher iron status, as well as significantly lower risk of IDA in the third trimester, independent of potential confounders including CRP.

ID and IDA are prevalent nutritional deficiency disorders during pregnancy worldwide. Therefore, early identification of risk factors for ID and IDA is essential to prevent the occurrence of ID and IDA. It has been shown that hepcidin is a key regulator of iron metabolism. As known in previous studies, hepcidin level would continue to fall throughout pregnancy to allow more iron to be released into plasma to meet maternal iron requirements and the needs of fetal growth and development (21). In the present study, we did observe a substantial decline of hepcidin from the first to the third trimester. Our finding is in accordance with previous findings that hepcidin undergoes apparent changes during pregnancy (18, 22). However, a prospective cohort study included 103 healthy Turkish women with normal pregnancies found no significant differences in hepcidin between early pregnancy and late pregnancy, probably because the effects of obstetrical complications such as anemia or ID on maternal hepcidin were not considered (23).

The association between hepcidin and iron status during pregnancy has been reported in previous researches (14, 17, 24). However, the relationships between hepcidin in early pregnancy and iron biomarkers in late pregnancy have rarely been analyzed. Of note, positive associations between first-trimester serum hepcidin and third-trimester serum hepcidin, Hb and SI were observed in our study. A plausible explanation is that although hepcidin decreases with the progress of pregnancy, hepcidin that is higher in the first trimester remains at a higher level in the third trimester, indicating a replete iron status, which partly counteracts suppression of hepcidin by pregnancy signals (9).

Numerous prior studies measured hepcidin during pregnancy when ID/IDA was already diagnosed (14, 15), whereas we investigated hepcidin of non-ID/IDA pregnant women in the first trimester and ID/IDA risk in the third trimester. We found that elevated first-trimester serum hepcidin was associated with diminished risk of third-trimester IDA, similar to some studies reporting a negative relationship between hepcidin and IDA (14, 15, 25). Previous studies have demonstrated that hepcidin inhibits iron efflux into plasma by degrading its only receptor, ferroportin (FPN) in hepatocytes, intestinal enterocytes, and macrophages, thereby leading to iron sequestration in cells (26). During iron replete pregnancy, maternal iron and hepcidin metabolism keep homeostatic, the mothers maintain constant SI levels and relatively high but not overexpressed serum hepcidin levels, despite increased iron utilization in advanced pregnancy. However, in iron-overloaded mothers, hepcidin production is overstimulated, resulting in hypoferremia and limiting the iron availability for both the mother and the fetus (9). SF is a stable and valid indicator reflecting iron stores, and it can be found in our study that first-trimester SF levels were higher among those women with higher first-trimester serum hepcidin levels but did not reach the threshold of iron overload. Therefore, a possible reason for our findings is that elevated hepcidin levels in early pregnancy implies replete iron stores, indicating that sufficient iron is available for Hb synthesize as pregnancy progresses, thereby decreasing IDA risks in the third trimester. Recently, Sangkhae et.al assessed maternal hepcidin suppression in different iron status using mouse models and found that compared with nonpregnant levels, hepcidin mRNA and protein levels in iron-replete pregnant mice were already almost suppressed at the earliest examined time point, whereas levels of liver iron were not yet largely decreased (9). The lowering of hepcidin is presumed to precede liver iron mobilization. This indicates that decline in hepcidin reflects a physiologic state of high iron requirement, although the onset of low iron stores may not yet occur at this time, when timely iron supplementation may effectively prevent the development of ID/IDA in late pregnancy (16). Except for iron stores, another important factor that affects hepcidin levels is anemia itself, through erythroferrone (ERFE). Studies have demonstrated that ERFE is a glycoprotein hormone secreted by erythroblasts in response to erythropoietin (EPO) stimuli such as hemorrhage, hypoxia, EPO therapy, β-thalassemia, and anemia of inflammation, and it suppresses the hepatic production of hepcidin, thereby mobilizing iron for erythropoiesis (27). Accordingly, it can be speculated in our study that lower levels of first-trimester serum hepcidin may imply a state of EPO-stimulated ERFE production, as well as a possible condition of high anemia risk, although all the participating women were below the diagnosis threshold of anemia at enrollment. Taken together, further studies is warranted to collect more information on iron storage indicators and erythroid regulatory factors, thereby elucidating the mechanisms underlying the association between higher levels of first-trimester serum hepcidin and lower risk of third-trimester IDA.

The relationship between hepcidin and ID was also investigated in the present study. No significant association was observed between first-trimester hepcidin and third-trimester ID risk, which is consistent with a Tanzanian study that found no relationship between baseline hepcidin (< 28 weeks) and ID at delivery (11). However, there are some studies with inconsistent findings (9, 11, 16). For example, an analysis among pregnant women based on an incorporative dataset of clinical trials and a prospective cohort found that hepcidin in individuals with ID was significantly lower than iron-replete individuals (11). Additionally, in another cohort study in Gambia, the prevalence of maternal ID increased, while hepcidin gradually decreased with progressive gestation (16). Furthermore, a follow-up study among iron-deficient Tanzania pregnant women taking iron supplements showed that the prevalence of ID dropped from 93 to 12%, while hepcidin increased form 1.0 μg/L at baseline to 12.3 μg/L at delivery (28). The discrepancy may be partially due to the fact that criteria to determine ID differ and that serum hepcidin levels are often undetectable or low in ID. Therefore, those inconsistent results between hepcidin and ID still call for further investigation.

The strengths in our study include a prospective cohort design and detailed information on potential confounders. We confirmed a negative association between first-trimester hepcidin and third-trimester IDA risk in Chinese population. Additionally, this is the first study to examine the associations between first-trimester hepcidin and third-trimester iron biomarkers within the Chinese setting. However, limitations should be acknowledged as followed. First, a major limitation in our study is the relatively small number of IDA women in each category of first-trimester serum hepcidin levels, and thus the EPV (events per variable) criterion was marginally met when performing logistic regression analysis. Given that pregnant women are usually advised a range of measures preventing ID/IDA (e.g., iron, folic acid and vitamin C supplementation) during routine pregnancy care, and that the study population was derived from a single hospital, it is not surprising that few women developed IDA in late pregnancy. Hence, although we revealed the potential negative association of first-trimester serum hepcidin with third-trimester IDA, our findings were mainly explorative and caution should be exercised when extrapolating to other groups. Second, the relationships between serum hepcidin levels in the second trimester and iron status in the third trimester were not evaluated in the present study. Finally, only CRP was collected in our study, yet hepcidin is affected by other inflammatory factors. Thus, further multi-center study with larger sample size is warranted to provide more evidence on the association between serum hepcidin and IDA during consecutive trimesters of pregnancy while taking into account the effects of other inflammatory factors.

## 5. Conclusion

Our study demonstrated that elevated first-trimester serum hepcidin level was closely associated with decreased risk of IDA in the third trimester, which indicates that high hepcidin level in early pregnancy implies replete iron stores and therefore lower risk of IDA in late pregnancy. However, considering the relatively limited sample size, larger studies collecting more data on iron metabolism indicators and inflammatory factors during consecutive trimesters are still needed.

## Data availability statement

The raw data supporting the conclusions of this article will be made available by the authors, without undue reservation.

## Ethics statement

The studies involving human participants were reviewed and approved by the Ethics Committee of School of Public Health, Sun Yat-sen University. The patients/participants provided their written informed consent to participate in this study.

## Author contributions

YaZ and PS designed the study. PS, SX, YuZ, XW, XL, HL, ZL, LZ, and FH enrolled the participants. SX and YaZ analyzed the data. PS, SX, and YuZ wrote the article. SX, YuZ, and YaZ interpreted the results. PS, XL, LZ, ZL, and FH contributed intellectually to the manuscript. YaZ had primary responsibility for final content. All authors have read and approved the final manuscript.

## Funding

The study was funded by the Guangdong Provincial Natural Science Foundation (Grant No. 2021A1515010439), medical science and technology of Guangdong Province (No. A2019332), and the Sanming Project of Medicine in Shenzhen (Grant No. SZSM201803061). The funders had no role in study design, data collection and analysis, decision to publish, or manuscript preparation.

## Conflict of interest

The authors declare that the research was conducted in the absence of any commercial or financial relationships that could be construed as a potential conflict of interest.

## Publisher’s note

All claims expressed in this article are solely those of the authors and do not necessarily represent those of their affiliated organizations, or those of the publisher, the editors and the reviewers. Any product that may be evaluated in this article, or claim that may be made by its manufacturer, is not guaranteed or endorsed by the publisher.
