# Supplementary figures and images for: Elevated first-trimester hepcidin level is associated with reduced risk of iron deficiency anemia in late pregnancy: a prospective cohort study
Source: Front Nutr. 2023 Aug 15;10:1147114. doi: 10.3389/fnut.2023.1147114 (PMC10465702; doi:10.3389/fnut.2023.1147114)

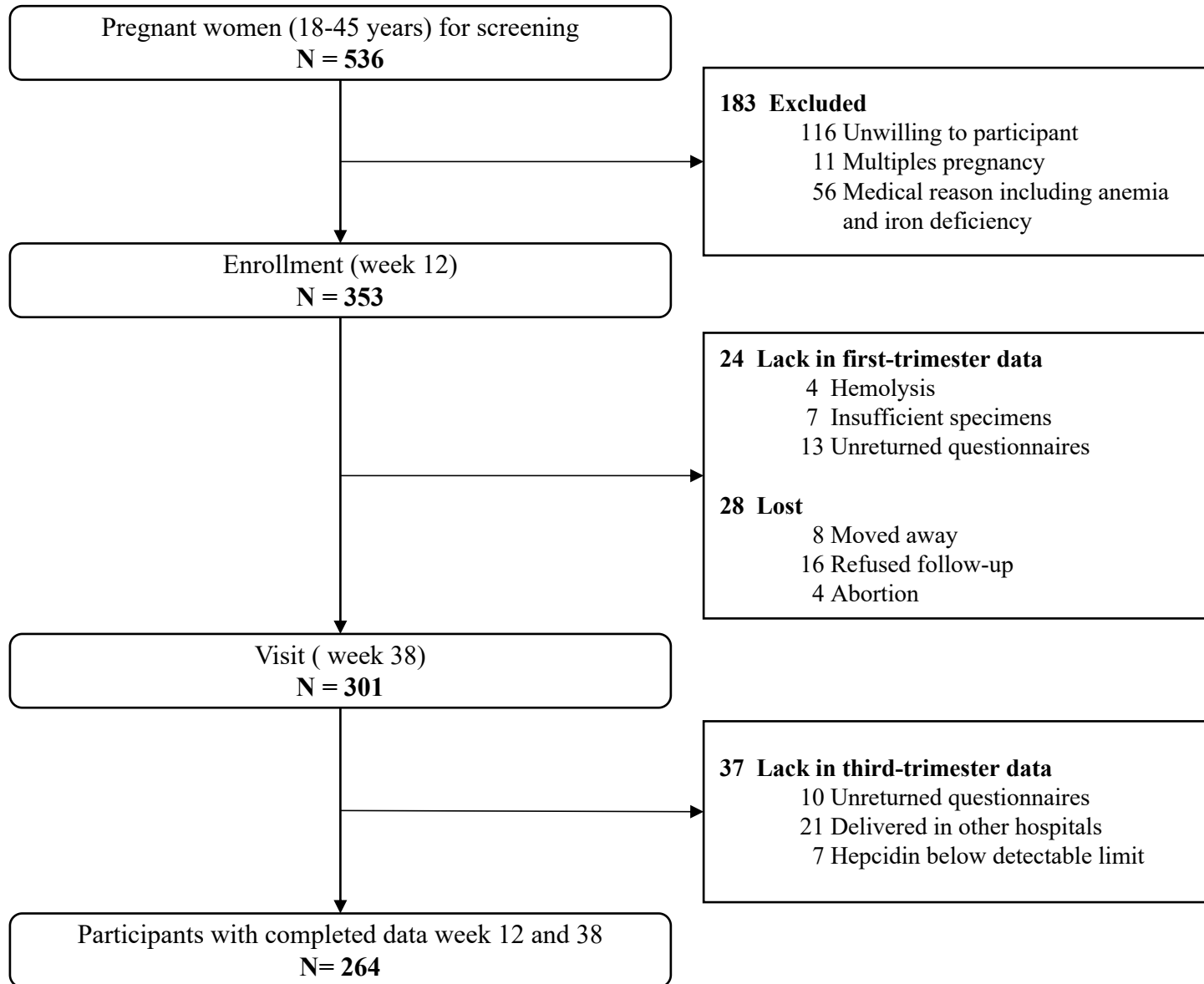

Supplementary Figure 1. Participants flow from recruitment through 38-week follow-up

Supplement: Supplementary file 1 [file Data_Sheet_1.PDF]
